# Fertility, Pregnancy, and Psychological Burden in OHVIRA Syndrome: Clinical Case Study and Review of the Literature

**DOI:** 10.3390/jcm15124806

**Published:** 2026-06-21

**Authors:** Natalia Katarzyna Mazur-Ejankowska, Zuzanna Małgorzata Brzóska, Maciej Ejankowski, Amelia Sztangierska, Kinga Jaguszewska, Dariusz Grzegorz Wydra, Magdalena Emilia Grzybowska

**Affiliations:** 1 Department of Gynecology, Obstetrics and Neonatology, Medical University of Gdansk, 80-210 Gdansk, Polandmagdalena.grzybowska@gumed.edu.pl (M.E.G.); 2 Clinic of Obstetrics and Gynecology, Gynecological Oncology and Endocrine Gynecology, University Clinical Centre, 80-952 Gdansk, Poland; 3 First Doctoral School, Medical University of Gdansk, 80-210 Gdansk, Poland; 4 Division of Medicine, Medical University of Gdansk, 80-210 Gdansk, Poland

**Keywords:** OHVIRA syndrome, Herlyn-Werner-Wunderlich syndrome, Müllerian duct anomaly, fertility-related anxiety, psychological burden, uterus didelphys, obstructed hemivagina, renal agenesis, mental health in gynecology

## Abstract

**Introduction:** Obstructed HemiVagina and Ipsilateral Renal Anomaly (OHVIRA) syndrome, also known as Herlyn–Werner–Wunderlich syndrome, is a rare congenital Müllerian duct anomaly, characterized by uterus didelphys, obstructed hemivagina, and ipsilateral renal agenesis. Symptoms typically appear shortly after menarche and include dysmenorrhea and pelvic pain. The psychological burden associated with fertility and reproductive outcomes in women with OHVIRA syndrome remains poorly investigated. **Materials and methods:** A 30-year-old primigravida with left renal agenesis and a history of vaginal abscess, dysmenorrhea, and chronic pelvic pain received a delayed OHVIRA syndrome diagnosis. The patient had previously been informed that spontaneous conception and an uncomplicated pregnancy were highly unlikely because of her congenital gynecological condition, resulting in significant fertility-related anxiety and psychological distress. Under careful supervision and counseling, she conceived successfully, and the pregnancy progressed without complications; an elective cesarean section was performed at term. A literature search using the PubMed and Embase databases was conducted between November 2025 to April 2026 to identify studies reporting reproductive outcomes and psychological aspects in patients diagnosed with OHVIRA syndrome and other Müllerian anomalies. **Results:** Evidence-based counseling contributed to improvement of quality of life and reduction of pregnancy-related anxiety of the reported patient with OHVIRA syndrome. A limited number of studies discuss the mental burden and fertility-related anxiety of patients with OHVIRA syndrome and other Müllerian anomalies. **Conclusions:** Spontaneous conception and uncomplicated pregnancy are possible for women with OHVIRA syndrome. The psychological burden associated with congenital gynecological conditions remains under-recognized and requires further investigation. Comprehensive counseling and interdisciplinary care are essential to improve reproductive education, mental health support, and pregnancy outcomes in patients with congenital gynecological anomalies.

## 1. Introduction

Obstructed HemiVagina and Ipsilateral Renal Anomaly (OHVIRA) syndrome, previously known as Herlyn–Werner–Wunderlich syndrome, is a rare congenital condition affecting the reproductive and the urinary systems. It is characterized by the classical triad of anomalies: uterus didelphys, obstructed hemivagina, and ipsilateral renal agenesis [1,2,3]. This congenital condition results from defects in the differentiation, fusion, and resorption of the Müllerian and Wolffian ducts during intrauterine fetal development [3,4]. Most commonly, the syndrome manifests shortly after menarche, in the early adolescence [1,2,3]. The symptoms include chronic pelvic pain and dysmenorrhea, which may be a result of the retention of menstrual blood in the obstructed hemivagina, leading to a hematocolpos or, less commonly, a hydrocolpos. Hence, a palpable pelvic or lower abdominal mass may be present in some OHVIRA patients upon a gynecological examination. Recurrent genital or systemic infections are typical in the lead up to OHVIRA syndrome diagnosis [4]. In sexually active patients, dyspareunia may be observed [4]. The nonspecific clinical presentation of OHVIRA syndrome may cause a delay in the diagnosis and some patients, despite their symptoms, are diagnosed later, including during pregnancy [5,6,7]. A late diagnosis in the fifth decade of life is also possible and has been reported [8].

Magnetic resonance imaging remains the golden standard for Müllerian ducts anomalies diagnosis [2]. Expert transvaginal and transabdominal ultrasound is also essential in the diagnostic process and should be treated as an effective first-line screening tool upon a suspicion of obstructive Müllerian anomalies [3]. It is crucial to underline that differences in the clinical presentation are common, and ipsilateral renal agenesis is not always diagnosed, and some authors argue that a change of the syndrome’s name to “ipsilateral renal anomalies” should be considered to highlight the range of urinary presentations [9]. It is key for the diagnostic process that the urological symptoms may include dysplastic and atrophic kidneys, which may not be easily visualized on any imaging studies [9]. Screening for Müllerian anomalies is recommended for females with prenatally detected renal abnormalities [1]. Prenatal diagnosis of OHVIRA syndrome is possible and should be considered in female fetuses with renal and urogenital abnormalities including renal agenesis and vaginal cysts [10] and should be followed up postnatally.

The etiology of OHVIRA syndrome is unknown and a genetic background cannot be excluded and is under investigation in relation to renal agenesis-related genes and UMOD (Uromodulin) gene variants, especially in patients with unexpected renal dysfunction, chronic kidney disease and hyperuricemia [11,12]. The incidence of all Müllerian anomalies is estimated at 0.1% to 3.8% [2]; however, it is most likely under-reported and underestimated due to limited knowledge of the condition and diagnostics challenges. Patients with Müllerian anomalies including OHVIRA syndrome face gynecological symptoms which can be resolved operatively; however, the implications of the condition usually continue and may disrupt future fertility and reproductive outcomes [13,14] and may cause a significant psychological burden. The OHVIRA syndrome may be incorrectly associated with infertility, due to limited clinical exposure of medical practitioners to the condition. Insufficient awareness regarding fertility in this group of patients may lead to misinformation. Such misconceptions may cause unnecessary psychological distress and may negatively affect reproductive decision-making, particularly in young women. Despite the adequate progression of the gestation, the longstanding belief in infertility might cause constant anxiety during this already demanding period for women.

The aim of the review is to assess the available literature on fertility-related psychological burden of women with OHVIRA syndrome and other Müllerian anomalies. To illustrate the importance of accurate reproductive counseling and pregnancy monitoring, a case study of a pregnant patient with OHVIRA syndrome is presented.

## 2. Materials and Methods

A case study of a pregnant patient with OHVIRA syndrome is presented with attention to the psychological burden and fertility-related anxiety. Written informed consent was obtained from the patient, including her medical history, laboratory and imagining results.

A literature search was performed by two researchers (N.M.E. and Z.B.) between November 2025 to April 2026 using the PubMed and Embase databases to identify studies about psychological burden related to fertility and pregnancy in OHVIRA syndrome patients. The used search terms included: OHVIRA syndrome, Herlyn–Werner–Wunderlich syndrome, Müllerian duct anomaly and fertility-related anxiety, psychological burden, mental health. No articles focusing specifically on the psychological burden of OHVIRA syndrome patients were identified. The available evidence consisted mainly of descriptive case-based reports and studies focusing on patients with other than OHVIRA syndrome Müllerian anomalies, predominantly Mayer–Rokitansky–Küster–Hauser syndrome (MRKH). The findings and limitations of the review were summarized narratively. Only studies reporting on the psychological burden and quality of life were included in the review.

### Case Presentation

A 30-year-old woman presented to the outpatient clinic due to fertility-related anxiety and to discuss the possibility of pregnancy planning. Her medical history was notable for congenital left renal agenesis diagnosed by transabdominal ultrasonography in early childhood. In adolescence, she began to experience several gynecological symptoms, including dysmenorrhea, menorrhagia, and recurring pelvic pain. These symptoms significantly impaired her daily functioning and quality of life, leading numerous medical visits and transabdominal ultrasonography scans which were performed without conclusive findings. At 20 years old, the patient was admitted to gynecological clinic, where an abscess on the left side of the vaginal wall was identified and surgically drained. During the surgical procedure, an obstructed hemivagina was discovered on the left side and the obstruction was surgically excised enabling future menstrual blood outflow. Intraoperatively uterus didelphys was identified, consisting of two completely separate hemiuteri, each with its own cervix uteri, without an intervening uterine septum. Following the procedure, the patient was informed that, because of this anatomical anomaly, spontaneous conception would be unlikely or even impossible. At this time an OHVIRA diagnosis was not made, possibly due to limited information about the condition, and the patient was not referred to another specialist.

**Figure 1:**
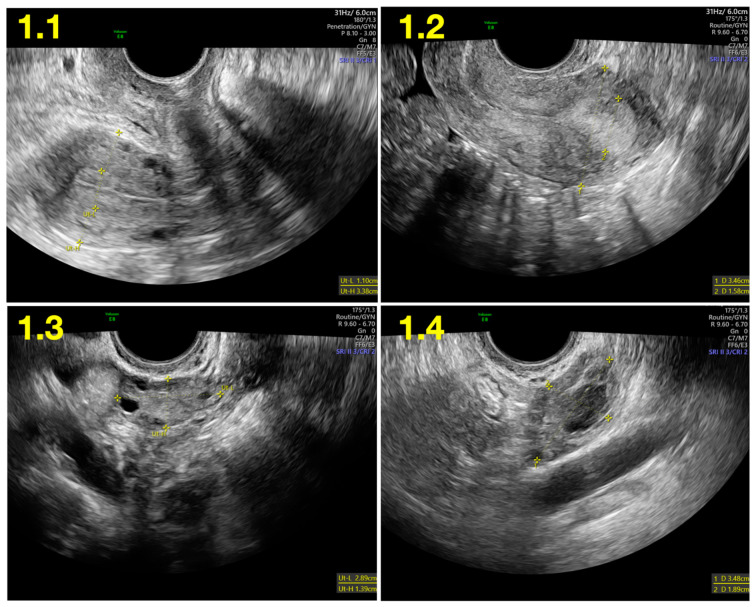
Transvaginal ultrasonography prior to conception illustrating uterus didelphys and ovaries. (**1.1**) Right uterus: distance 1 (Ut–L on image)—endometrial thickness 1.10 cm; distance 2 (Ut–H on image)—uterus height 3.38 cm. (**1.2**) Left uterus: distance 1 (1D on image)—uterus height 3.46 cm; distance 2 (2D on image)—endometrial thickness 1.58 cm. (**1.3**) Left ovary with normal morphology and size of 29 × 14 mm. (**1.4**) Right ovary with normal morphology and size of 35 × 19 mm.

A decade later, during a routine gynecological consultation with a new gynecologist, transvaginal ultrasonography was performed. The examination confirmed the presence of uterus didelphys and two ovaries which were normal in morphology and size (Figure 1). Combination of the patient’s medical history including the previously obstructed left hemivagina and left renal agenesis which was confirmed by transabdominal ultrasonography led to the diagnosis of OHVIRA syndrome. During clinical interview the patient reported extensive fertility-related anxiety, based on her previous fertility prognosis received from other medical practitioners.

**Figure 2:**
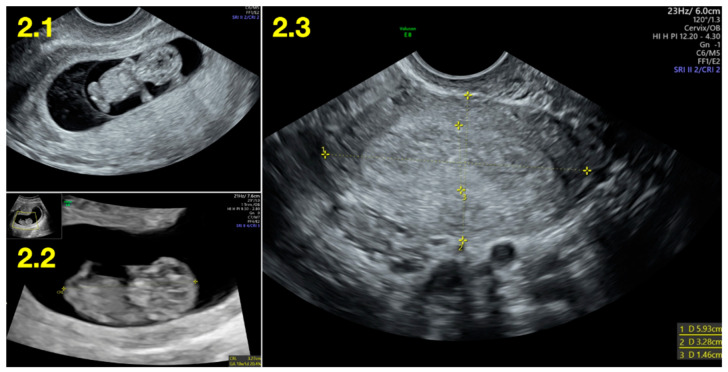
Transvaginal ultrasonography images at 10 weeks and 1 day of pregnancy. (**2.1**,**2.2**). Left uterus with a gestational sack and fetus at 10 weeks and 1 day of intrauterine development, Crown-Rump Length (CRL) was 3.27 cm. (**2.3**) Right uterus: distance 1 (1D on image)—uterus length 5.93 cm; distance 2 (2D on image)—uterus height 3.28 cm; distance 3 (3D on image)—endometrial thickness 1.46 cm.

**Figure 3:**
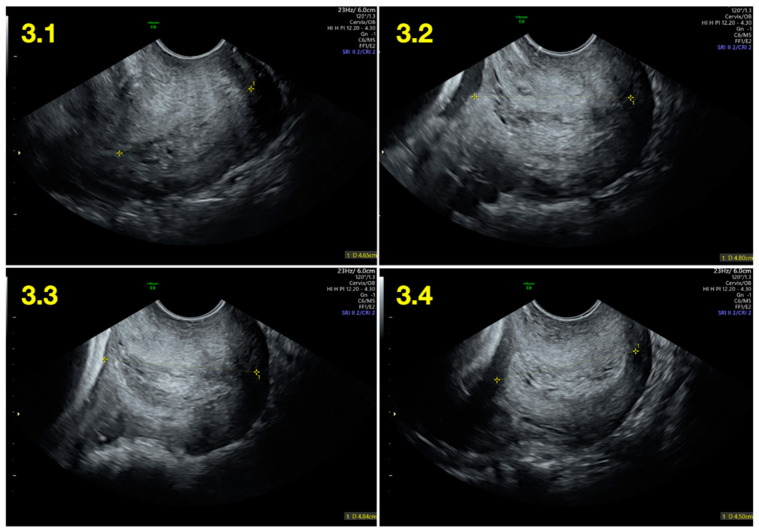
(**3.1**) Cervical length at 15 weeks and 2 days of gestation was 4.65 cm (1D on image—distance 1). (**3.2**) Cervical length at 28 weeks and 2 days of gestation was 4.80 cm (1D on image—distance 1). (**3.3**) Cervical length at 30 weeks and 2 days of gestation was 4.84 cm (1D on image—distance 1). (**3.4**) Cervical length at 32 weeks and 2 days of gestation was 4.50 cm (1D on image—distance 1).

The patient was counseled and informed that fertility is preserved in most women with OHVIRA syndrome and that pregnancy is of a high risk, but is not contraindicated. This intervention had a significant positive impact on her mental well-being and decision-making about future fertility plans. Within the next months, she conceived spontaneously without any fertility treatments. Transvaginal ultrasound examination confirmed a viable pregnancy located in the left uterus and a right uterus without abnormal findings (Figure 2). The pregnancy was closely monitored and progressed without any maternal or fetal complications. Regular ultrasound examinations confirmed normal fetal growth, normal amniotic fluid volume and normal placental development. Cervical length was regularly screened by transvaginal ultrasonography and remained within standard length limits throughout the pregnancy (Figure 3). No episodes of vaginal bleeding or symptoms of preterm labor were recorded. No hospitalization during the pregnancy was required. The patient’s mental status was assessed throughout the pregnancy using Beck’s Depression Inventory and the score did not reveal depression. Additionally, the patient self reported lower anxiety levels.

**Figure 4:**
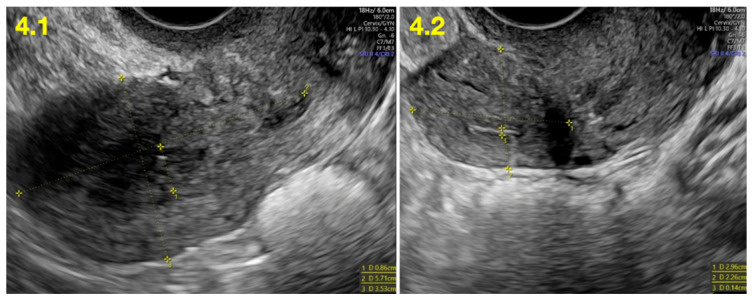
Transvaginal ultrasonography images illustrating postpartum assessment at 6 weeks after cesarean section. (**4.1**) Left uterus: distance 1 (1D on image)—endometrial thickness 0.86 cm; distance 2 (2D on image)—uterus length 5.71 cm; distance 3 (3D on image)—uterus height 3.53 cm. (**4.2**) Right uterus: distance 1 (1D on image)—uterus length 2.96 cm; distance 2 (2D on image)—uterus height 2.26 cm; distance 3 (3D on image)—endometrial thickness 0.14 cm.

**Figure 5:**
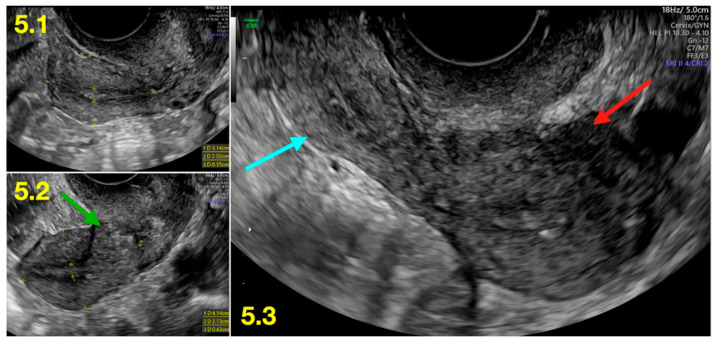
Transvaginal ultrasonography images at 6 months after cesarean section. (**5.1**) Right uterus, distance 1 (1D on image)—uterus length 3.14 cm, distance 2 (2D on image) uterus height 2.02 cm, distance 3 (3D on image)—endometrial thickness 0.35 cm. (**5.2**) Left uterus with a healed cesarean section scar (green arrow), distance 1 (1D on image)—uterus length 3.14 cm, distance 2 (2D on image) uterus height 2.02 cm, distance 3 (3D on image)—endometrial thickness 0.35 cm. (**5.3**) A normal post-pregnancy involution of uterus didelphys (blue arrow right uterus, red arrow left uterus).

At 40 weeks of gestation, the patient was admitted to a tertiary care center for delivery. Elective cesarean section was opted for after individualized obstetric assessment due to the uterus malformation and risk of inappropriate uterine contractility. A cesarean section on the left uterus was performed; the normal-sized right uterus was left intact during the procedure. A healthy male neonate weighing 3230 grams was delivered, with Apgar scores of 10/10/10 at 1, 5, and 10 minutes, respectively. After birth, the neonate presented with mild left-sided positional asymmetry, with was related to the intrauterine position in the left uterus; it was managed conservatively with physiotherapy. The postpartum period was uneventful apart from typical abdominal pain corresponding to the incision site in the left uterus which was successfully managed by oral Non-Steroidal Anti-Inflammatory Drugs (NSAIDs). At the postpartum visit 6 weeks after cesarean section a transvaginal ultrasound revealed a uterine scar with normal residual myometrial thickness during the standard healing process and a normal size of the right uterus (Figure 4). Six months after the cesarean section, a transvaginal ultrasound performed during a follow-up visit confirmed a healed uterine scar in the left uterus, normal endometrial thickness in the left and right uterus and a normal post-pregnancy involution of uterus didelphys (Figure 5).

## 3. Results and Discussion

### 3.1. Fertility and Pregnancy Outcomes in OHVIRA Syndrome

The data about the rates of fertility and infertility in OHVIRA syndrome remains limited. One of the biggest studies by Liu et al. consists of a literature review of 1673 cases of women in which OHVIRA syndrome was reported with a pregnancy rate of 72.1% [15]. Pregnancy of patients with OHVIRA syndrome is high risk and examination and monitoring by experienced obstetricians or maternal–fetal practitioners ensures early recognition of conditions such as fetal growth restriction and cervical insufficiency which are more common in this group of patients [13,15,16,17].

A study by Panitescu et al. [16] reports that fertility is preserved in women with OHVIRA syndrome and that there are conflicting results regarding the incidence of miscarriage and the rate of preterm delivery, which is estimated at 15–22%. The rate of cesarean section in OHVIRA syndrome patients is high at around 80%. The authors underline that the patients required psychological support to avoid anxiety and emotional disorders related to sexual quality of life and reproductive outcomes.

One of the largest studies by Erkkinen et al. [17] included 23 pregnancies among eight OHVIRA syndrome patients resulting in 9 term births, 6 preterm births, and 8 first-trimester spontaneous abortions. The most common complications were fetal growth restriction and preeclampsia with severe features, which occurred in 2 out of 15 pregnancies. The authors reported an average gestational age at delivery as 37 weeks and 2 days. It is crucial to underline that 60% of live births were at term. Two patients delivered after going into preterm labor, one of whom also had preterm premature rupture of membranes. There were 10 cesarean deliveries, 4 vaginal deliveries, and 1 forceps-assisted vaginal delivery in the group of 15 live births. Three patients underwent primary cesarean deliveries due to fetal malpresentation which is commonly reported in pregnancies of OHVIRA syndrome patients.

A case series of three patients after OHVIRA surgery by Bunnell et al. [13] demonstrated generally favorable pregnancy outcomes with successful term and near-term pregnancies. The authors discussed a number of clinical risks that are important for patient–provider counseling including: recurrent miscarriage, malpresentation, postpartum hemorrhage, retained placenta, fetal growth restriction, preterm birth, and premature rupture of membranes.

### 3.2. The Psychological Impact of Müllerian Anomalies

Misconceptions about fertility and pregnancy outcomes and inaccurate patient counseling may cause unnecessary psychological distress and may negatively affect reproductive decision-making in women with OHVIRA syndrome and should be avoided.

The literature search did not identify any studies specifically focusing on the psychological burden and fertility- and pregnancy-related anxiety in patients with OHVIRA syndrome. A number of studies discussing the psychological wellbeing of patients with other congenital Müllerian anomalies was identified. Out of other Mullein anomalies, MRKH syndrome was the most commonly investigated in relation to psychological implications. Tsarna et al. [18] investigated the impact of MRKH syndrome on the quality of life, psychological wellbeing and sexual life of patients. The authors concluded that MRKH could be associated with a higher prevalence of anxiety and depression symptoms compared with patients of a similar age without the condition. Additionally, the review specifically noted that diagnosis often occurs during adolescence, when sexual identity and body image are developing which could lead to greater impairment of mental health-related quality of life. The infertility-related emotional burden was also observed in patients with the condition [18]. Another study by Facchin et al. [19] underlines that MRKH syndrome may be associated with psychological symptoms, impaired quality of life, and poor sexual esteem and genital image, leading to patients experiencing difficulties during intimacy. Those findings can be relevant to the psychological distress experienced by OHVIRA patients, who also, most commonly, receive the diagnosis during early adolescence and who are also faced with altered gynecological anatomy.

A large cross-sectional study of anxiety in patients with MRKH revealed severe anxiety symptoms in 24.1% of patients [20]. The findings highlight the importance of anxiety symptoms screening in patients with congenital gynecological conditions to identify patients who should be counseled and treated to alleviate their mental health symptoms. Additionally, coexisting depressive symptoms were associated with increased onset of anxiety symptoms in patients with MRKH syndrome, which is in line with other studies reporting about comorbidity of anxiety and depression [20,21,22].

A recent qualitative study by Aitelli et al. [23] investigated sexual function in women with Müllerian anomalies. The authors concluded that patients face unique sexual challenges due to their anatomy and patient education materials and screening tools specific to this population are needed. These findings are relevant to patients with OHVIRA syndrome, who could benefit from evidence-based education and counseling from the diagnosis and throughout lifetime.

The prevalence of anxiety in infertile women is high and it affects quality of life and requires serious consideration and planning for effective intervention [24]. The study by Kiani et al. [24] indicated that 36% of the infertile women included in the meta-analyzes had anxiety symptoms and women from low and middle income countries were most affected. High prevalence of depressive and anxiety disorders in women undergoing assisted reproductive techniques has been confirmed by Chen et al. [25] and 40.2% of studied women had a psychiatric disorder. Generalized anxiety disorder was the most common, affecting 23.2% of patients, followed by major depressive disorder affecting 17.0% of patient, and dysthymic disorder affecting 9.8% of women. Importantly, women with a psychiatric morbidity did not differ from those without in terms of age, education, income, or years of infertility. Additionally, women with a history of previous assisted reproduction treatment did not differ from those without in depression or anxiety. Those results highlight the high risk of anxiety and depression in women with fertility problems and support early mental health intervention and support for women at risk of infertility. This is in line with Tomecka et al.’s [26] conclusions which underline that early surgical intervention in symptomatic OHVIRA patients increases fertility chances, and as a result, the quality of life increases.

### 3.3. Screening for Müllerian Anomalies

Screening for Müllerian anomalies is recommended for females with prenatally and postnatally detected renal abnormalities [1,27]. Fei et al. conducted a study of women in renal abnormalities (RA) and 38% of patients with RA who underwent a pelvic evaluation were found to also have Müllerian anomalies. The study descried the strongest association between solitary kidney and Müllerian anomalies. Importantly, about 40% of the patients who were diagnosed with Müllerian anomalies were found to have a vaginal obstruction requiring urgent treatment. The authors concluded that a delay in diagnosis and treatment of Müllerian obstructions can have a negative effect on future reproductive health, including increasing the risk of chronic pain, infertility, infection, and endometriosis. The findings led to a recommendation that in patients with RA, routine screening with pelvic ultrasound should be performed around the age of expected menarche [27]. This recommendation is supported by Walawender et al. [28] who conducted a study which identified Müllerian anomalies in 24% of children with congenital solitary functioning kidney and by Friedman et al. [29] who reported 30% prevalence of Müllerian anomalies in patients with unilateral renal agenesis.

Screening for Müllerian anomalies is crucial not only in patients with known urinary congenital conditions and primary amenorrhea, but also in symptomatic patients presenting with dysmenorrhea cyclic and chronic pelvic pain with and without menstrual abnormalities [30,31]. Fontana et al. conducted a study of 143 nomo-menstruating outpatients referred for severe dysmenorrhea and persistent pelvic pain and 42 young women (29.3%) were diagnosed with obstructive Müllerian anomalies irrespective of regular menstrual flow.

### 3.4. Limitations

The literature search did not identify any studies focusing on the psychological condition of women with OHVIRA syndrome and the search was expanded to include reports of MRKH syndrome, which also is a Müllerian duct abnormality. It should be underlined that MRKH syndrome is characterized by the absence of the upper part of vagina and a variable uterine development [15] and pregnancy in those patients is possible after a uterine transplantation [32].

Women diagnosed with OHVIRA syndrome may receive diagnosis of infertility without justification, as was presented in the case section of this article, which may lead to similar self-assessment as in patients with MRKH who are faced with infertility. The main limitation of this comparison is the difference in possible fertility prognosis.

Another limitation which should be acknowledged is the possibility of publication bias, as the majority of published studies describe successful pregnancy outcomes in women with OHVIRA syndrome. Adverse reproductive outcomes and the associated psychological issues are rarely reported, which may lead to an overly optimistic interpretation of the available evidence. This factor may be considered the greatest limitation of this study.

It should be underlined that Beck’s Depression Inventory was not performed before or after the pregnancy, which makes it challenging to objectively assess the patients’ psychological well-being.

## 4. Conclusions

OHVIRA syndrome patients experience altered gynecological and urinary anatomy which may lead to chronic gynecological symptoms including dysmenorrhea and pelvic pain and acute gynecological conditions of vaginal wall abscess and hematocolpos. Longterm consequences of the condition may include impaired fertility and complications during pregnancies, predominantly leading to increased risk of preterm birth. It is key to underline that spontaneous conception and uncomplicated pregnancy are possible for women with OHVIRA syndrome. Treatment of patients with OHVIRA syndrome should include interdisciplinary care with input from psychologists and psychiatrists to manage fertility- and pregnancy-related anxiety. Comprehensive counseling is essential to improve reproductive education and mental health support in patients with Müllerian anomalies.

The case study highlights that OHVIRA syndrome diagnosis may occur later in life due to limited awareness and knowledge of medical practitioners about the condition. Patients with gynecological and urinary abnormalities should be referred to a specialist center where medical practitioners with experience in congenital reproductive anomalies provide interdisciplinary care and evidence-based counseling. The pregnancy, delivery and postpartum care of women with OHVIRA syndrome should be provided by experienced obstetricians due to a high risk of complications.

## Data Availability

No new data were created or analyzed in this study.

## Abbreviations

MRKH	Mayer-Rokitansky-Küster-Hauser syndrome
NSAIDs	Non-Steroidal Anti-Inflammatory Drugs
OHVIRA	Obstructed HemiVagina and Ipsilateral Renal Anomaly syndrome
RA	Renal abnormalities
UMOD	Uromodulin
